# Supplementary material for: Data-driven organic solubility prediction at the limit of aleatoric uncertainty
Source: Nat Commun. 2025 Aug 19;16:7497. doi: 10.1038/s41467-025-62717-7 (PMC12365040; doi:10.1038/s41467-025-62717-7)
Supplement: Supplementary file 1 — Supplementary Information [file 41467_2025_62717_MOESM1_ESM.pdf]

Supporting Information: Data-driven Organic Solubility Prediction at the Limit of Aleatoric Uncertainty

Lucas Attia[1†], Jackson W. Burns[1†], Patrick S. Doyle[1], William H. Green[1*]

[1]Department of Chemical Engineering, MIT, Cambridge, MA.

*Corresponding author(s). E-mail(s): whgreen@mit.edu;
†These authors contributed equally to this work.

S1 Training and hyperparameter optimization details

The PyTorch [1] library is used to implement networks via the PyTorch Lightning [2] framework, which delivers excellent reproducibility and reusability. Network hyperparameters are optimized automatically using the Optuna software package [3]. During optimization, a holdout validation set was used to select ideal hyperparameters without restrictions on network size or expressiveness, ensuring that the maximum possible performance was achieved. Network hyperparameter optimization and training took place on the MIT SuperCloud High Performance Computing cluster[4] GPU nodes containing 2 x Nvidia Volta V100s. All reported metrics are defined according to their usual formulae and are implemented via standard Python machine learning packages. Early stopping was used during training, which allows the network to continue training until the error on the validation set increased, indicating overfitting. At that point, the network is reverted to the previous weights prior to the increase and carried forward for testing. The complete space of hyperparameters explored in the study is given in Table S1.

All code implementing the above is open source, permissively licensed, and available online through Ref [5].

Hyperparameter	Values
Input Activation	{sigmoid, tanh, clamp3}
Activation Function	{relu, leakyrelu, sigmoid, tanh}
Interaction Operation	{concatenation, hadamard product, sum, cartesian product}
Hidden Size	{400, 600, 800, ..., 3400}
Number of Layers	{0, 1, 2, ..., 6}
Solvent Hidden Size	{400, 600, 800, ..., 3400}
Solvent Layers	{0, 1, 2, ..., 6}
Solute Hidden Size	{400, 600, 800, ..., 3400}
Solute Layers	{0, 1, 2, ..., 6}

Table S1 Explored Hyperparameter Space

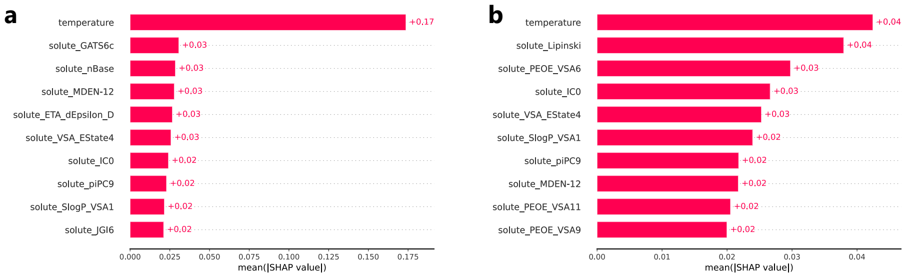

Supplementary Figure S1 Comparison of SHAP contribution plots for the FASTSOLV model on SolProp (a) and Leeds (b). The vertical axis gives the name of the input feature and the horizontal axis indicates the mean absolute SHAP value for each feature across all of the tested points For (a) 1000 test points were randomly selected and then SHAP was conditioned on 500 points and calculated on the remaining half. For (b) half of the entire dataset was used for conditioning, with the reminder used for calculating SHAP values. Source data are provided as a Source Data file.

S2 fastsolv SHAP analysis

As mentioned in the main text it is possible to use SHAP values [6] on our FASTSOLV model to quantify the importance of individual input features and thereby interpret model predictions. Said importances can also be used to deduce how many of the inputs features are required to achieve accurate predictions. This is epecially important because it is known that many molecular descriptors are highly correlated (e.g. molecular weight and count of common functional groups). Using the SHAP package, Figure S1 was created to address these points. The code for generating these plots is available alongside all other paper code on Github at github.com/JacksonBurns/fastsolv.

As shown the two test sets both use six of the same input features in their top ten, indicating that the representation learned on the training set is broadly applicable to chemical space and not degenerate with respect to other descriptor combinations. As further evidence of this, the following additional metric can be calculated: the number of features with an importance greater than the sum of all subsequent less-important features. For the SolProp and Leeds tests sets this number is 2,510 out of 3227 and

2,211 our of 3227, respectively. In each case, approximately 70% of the available features are used by the model. While feature selection could possibly eliminate some of the remaining 30% without a significant performance loss, the resuting model will still be quite large.

There are other notable features of these plots that are worth mentioning. Temperature goes down in importance when all tested point are at room temperature, as shown when comparing the SolProp (variable temperature) and Leeds (room temperature) datasets. This is an intuitive result given that solubility is known to be a strong function of temperature but is nonethless a good consistency check. Also important is that the top ten features are dominated by solute descriptors, rather than those of the solvent. This aligns with the physical understanding that solute chemical space is much broader than solvent chemical space. The diversity in structures in the former is well represented in the training data and thus learned by the model, whereas the limited number of industrial solvents limits both solvent diversity in the training data as well as actual applicable solvents in real world settings.

S3 Predictions on specific solutions

Furthermore, we evaluated the performance of FASTSOLV on other solutes of interest, namely anthracene and anthraquinone derivatives. First, within the SolProp dataset, we extracted all of the solutions of these solutes, compiling 85 anthracene, 2-ethylanthraquinone, and 1-chloroanthraquinone solutions in 53 unique solvents, all at room temperature. The test of FASTSOLV on this SolProp subset yields an overall RMSE = 0.52, with RMSE = 0.76 for 4 anthracene solutions, RMSE = 0.44 for 32 2-ethylanthraquinone solutions, and RMSE = 0.55 for 49 1-chloroanthraquinone solutions. These results suggest the model is highly accurate on these potentially challenging polycyclic aromatic compounds.

However, to extend this analysis to temperature-dependent solubility, we searched the literature for a temperature-dependent dataset of anthracene/anthraquinone solubility, finding the work of Cepeda and Diaz. These experimental results are plotted below against the FASTSOLV prediction curves in Supplementary Figure S2. We observe highly accurate anthracene predictions, with RMSE ranging between 0.12-0.42 for the different solvents. We also observe that the model accurately captured the temperature-dependence of anthracene solubility (Supplementary Figure S2a-c). In contrast, we observe less accurate predictions for anthraquinone solubility, with RMSE ranging between 1.20-1.80 for the different solvents (Supplementary Figure S2d-f). We do still observe accurate temperature gradients. Interestingly, the model uncertainty was much larger for the anthracene predictions than for anthraquinone predictions, despite the predictions being much more accurate. Overall, FASTSOLV demonstrates highly accurate performance on room-temperature anthracene, 2-ethylanthraquinone, and 1-chloroanthraquinone solutions within the SolProp test set, highly accurate predictions of temperature-dependent anthracene solutions, and relatively inaccurate predictions on temperature-dependent anthraquinone solutions.

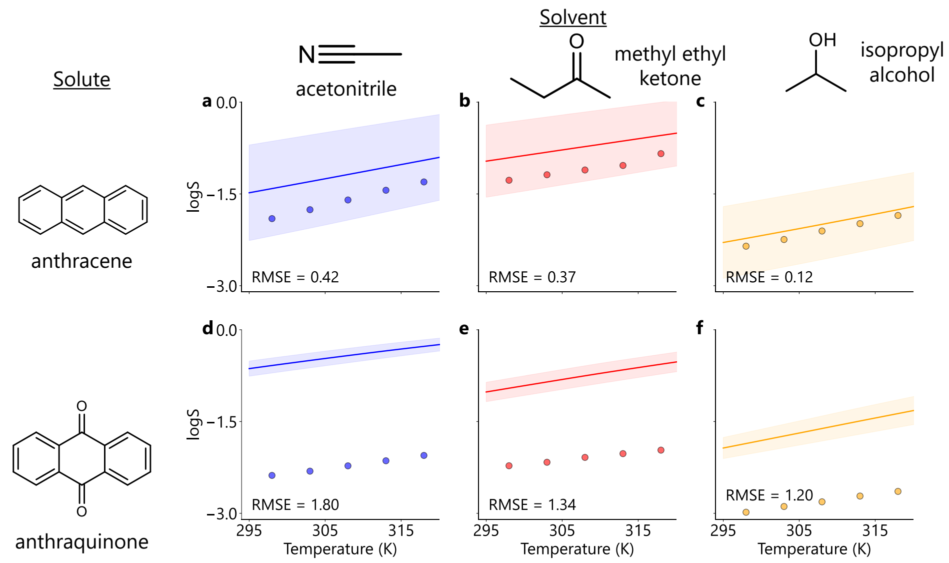

Supplementary Figure S2 FASTSOLV performance on anthracene and anthraquinone in various solvents. Solubility ($\log S$) predictions and experimental data as a function of temperature (K) for solutions of anthracene (top row) in acetonitrile (a), methyl ethyl ketone (b), and isopropyl alcohol (c); and anthraquinone (bottom row) in the same solvents (d-f). Experimental data are plotted as circles, while FASTSOLV predictions are plotted as solid lines. The shaded areas indicate model uncertainty, which is the ensemble standard deviation. Experimental solubility data is compiled from Cepeda and Diaz. Root mean square error (RMSE) for each solution is shown on the plot. Source data are provided as a Source Data file.

S4 Determination of aleatoric limit

As discussed in Section 1.3, we evaluated an estimate of the inter-laboratory experimental variability by comparing the experimental data from different solutions with the same solute, solvent, and temperature (within a threshold of 1° K), but different original sources between the different datasets. This mirrors the task of training on one dataset (BigSolDB), and testing extrapolating to another dataset (Leeds, SolProp). We found 34 solutions with different sources, containing 8 unique solutes and 6 unique solvents. The average inter-laboratory standard deviation between these solutions is 0.34 while the RMSE is 0.75. We then evaluated model predictions on these solutions with multiple sources. Specifically, measurements of N-acetylglycine solubility in acetonitrile and methanol from Zhao et al. and Guo et al. These two solutions are especially interesting case studies, since in acetonitrile, the experimental data differ drastically, but in methanol the data from both sources are very similar (Figure S3). For acetonitrile, the model predicts solubility values in good agreement with the measurements from Zhao et al., while in methanol the model predictions are in good agreement with both experiments. The fact that the model matches one of the experimental datasets in the case when they disagree is encouraging, particularly since that source indicates that the solubility should be higher in acetonitrile than in methanol,

which matches an intuitive chemical understanding because the solute contains an acetyl backbone.

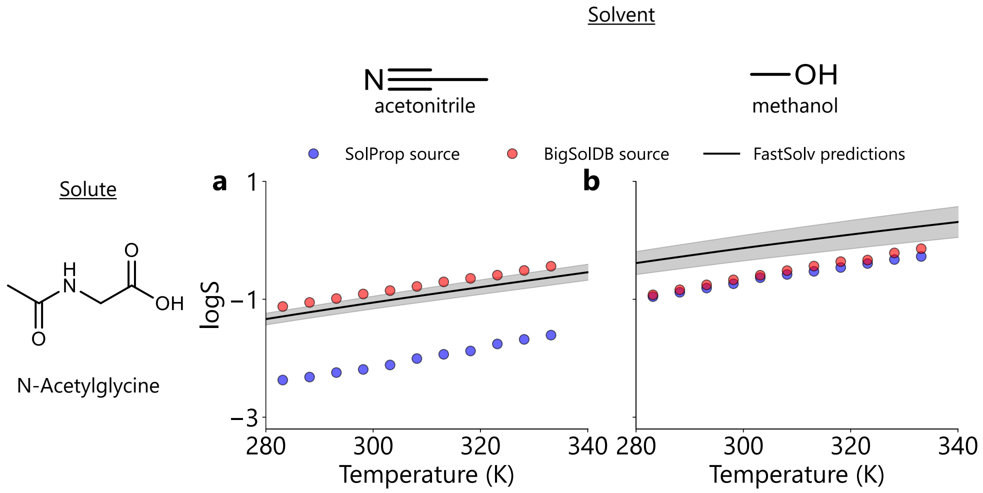

Supplementary Figure S3 Model performance on variable experimental data. Solubility of N-acetylglycine in (a) acetonitrile and (b) methanol. Red points show experimental data from Zhao et al., while blue points show experimental data from Guo et al. Solid line shows FASTSOLV model predictions. The gray shaded area represents the model uncertainty which is the ensemble standard deviation. Source data are provided as a Source Data file.

S5 Benchmarking against transformer-based molecular foundation models

We sought to determine whether the plateau in test RMSE reported in Figure 5 was attributable to epistmetic uncertainty from insufficiently expressive or otherwise erroneous models. To this end, we benchmarked our models against the state-of-the-art trasnformer-based molecular foundation models MolFormer[10] and ChemBERTa-2.[11] These widely-utilized transformer-based models have been found to perform extremely well at molecular property prediction tasks, and encode rich molecular embeddings which can be fine-tuned to specific tasks.[10] Critically, they take a fundamentally different approach to molecular modeling than either FASTPROP or CHEMPROP, using SMILES string directly rather than graphs or molecular descriptors.

We fine-tuned these models on the same training set from BigSolDB, using training protocols in tutorials provided by the original authors. The fine-tuned models were then tested on the SolProp and Leeds test sets, as highlighted below in Figure S4. We observe that MolFormer outperforms ChemBERTa on both the Leeds and SolProp test sets, but still underperforms compared to both of the models developed in the present study.

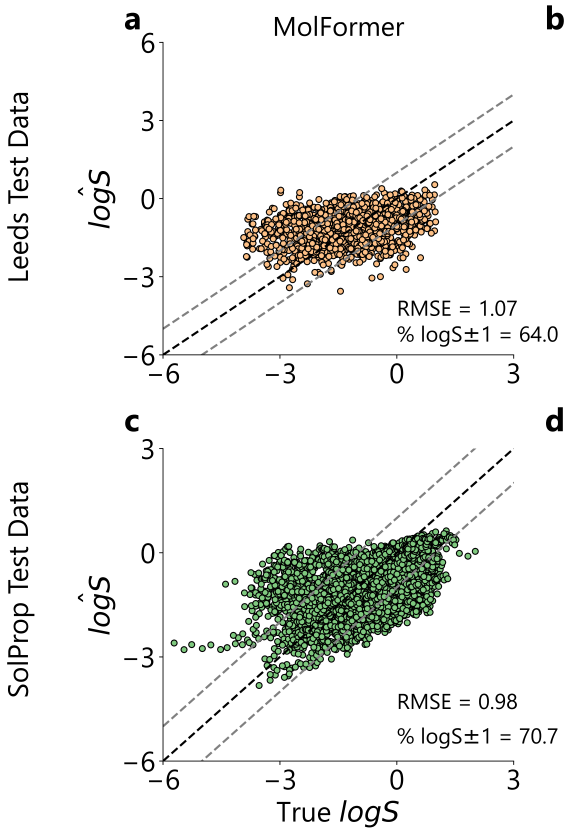

Supplementary Figure S4 Benchmarking against transformer-based models. Parity plots demonstrating ground truth solubility ($\log S$) against predicted solubility ($\log \hat{S}$) for fine-tuned MolFormer[10] (a and c) and ChemBERTa[11] (b and d) on the Leeds[12] (top row, a and b) and SolProp[13] (bottom row, c and d) test sets. The root mean square error (RMSE) and $\% \log S \pm 1$ are listed for each model on each test set. Source data are provided as a Source Data file.

S6 Solute distribution over chemical space

We used the chemplot package[14], to visualize the distribution of solutes in the training and testing data over chemical space. The 2-D UMAP plot below, in Figure S5 shows the broad coverage of solutes in the Leeds dataset compared to the narrower coverage of the solutes in the BigSolDB training data and SolProp testing data.

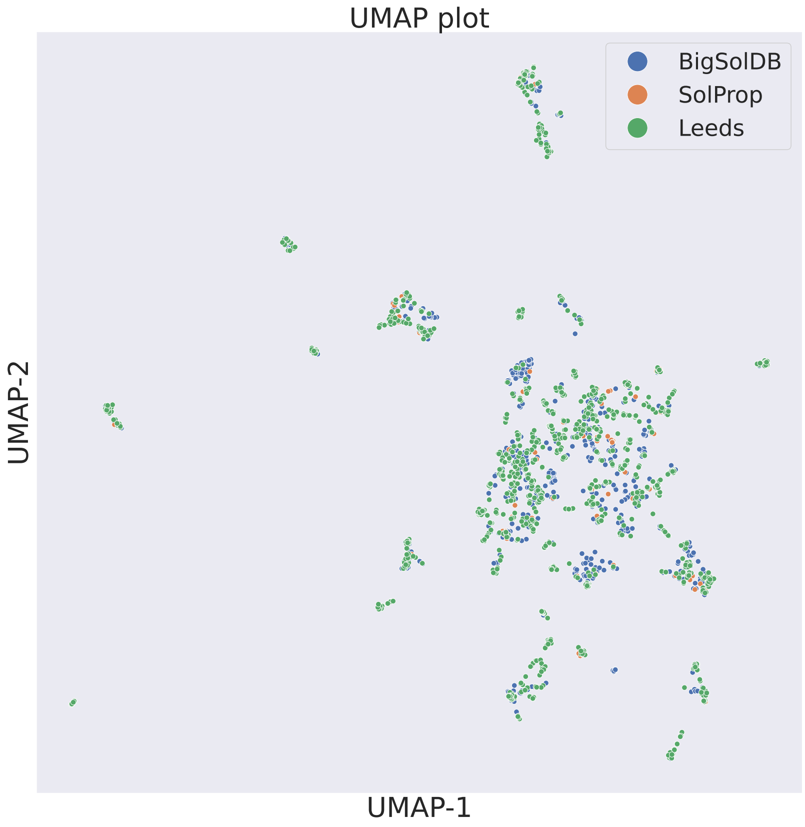

Supplementary Figure S5 Distribution of solutes in chemical space. 2-D UMAP projection of solute chemical space. Solutes from BigSolDB[15] (blue), SolProp[13] (orange), and Leeds[12] (green) are represented as points. Source data are provided as a Source Data file.

References

[1] Paszke, A., Gross, S., Massa, F., Lerer, A., Bradbury, J., Chanan, G., Killeen, T., Lin, Z., Gimelshein, N., Antiga, L., Desmaison, A., Köpf, A., Yang, E., DeVito, Z., Raison, M., Tejani, A., Chilamkurthy, S., Steiner, B., Fang, L., Bai, J., Chintala, S.: PyTorch: An Imperative Style, High-Performance Deep Learning Library (2019). https://arxiv.org/abs/1912.01703

[2] Falcon, W., The PyTorch Lightning team: PyTorch Lightning. https://doi.org/10.5281/zenodo.3828935 . https://github.com/Lightning-AI/lightning

[3] Akiba, T., Sano, S., Yanase, T., Ohta, T., Koyama, M.: Optuna: A next-generation hyperparameter optimization framework (2019) arXiv:1907.10902 [cs.LG]

[4] Reuther, A., Kepner, J., Byun, C., Samsi, S., Arcand, W., Bestor, D., Bergeron, B., Gadepally, V., Houle, M., Hubbell, M., Jones, M., Klein, A., Milechin, L., Mullen, J., Prout, A., Rosa, A., Yee, C., Michaleas, P.: Interactive supercomputing on 40,000 cores for machine learning and data analysis. In: 2018 IEEE High Performance Extreme Computing Conference (HPEC), pp. 1–6 (2018). IEEE

[5] Burns, J., Attia, L.: Data-driven Organic Solubility Prediction at the Limit of Aleatoric Uncertainty. Zenodo (2025). https://doi.org/10.5281/zenodo.15531356

[6] Vassileiou, A.D., Robertson, M.N., Wareham, B.G., Soundaranathan, M., Ottoboni, S., Florence, A.J., Hartwig, T., Johnston, B.F.: A unified ml framework for solubility prediction across organic solvents. Digital Discovery **2**(2), 356–367 (2023)

[7] Cepeda, E.A., Diaz, M.: Solubility of anthracene and anthraquinone in acetonitrile, methyl ethyl ketone, isopropol alcohol and their mixtures. Fluid phase equilibria **121**(1-2), 267–272 (1996)

[8] Zhao, W., Yang, W., Guo, Q., Fan, S., Hao, J., Hu, Y.: Thermodynamic models for determination of the solubility of n-acetylglycine in (methanol+acetonitrile) binary solvent mixtures. Journal of Thermodynamics & Catalysis **6**(2), 1000166 (2015) https://doi.org/10.4172/2157-7544.1000166

[9] Guo, Y., He, H., Huang, H., Qiu, J., Han, J., Hu, S., Liu, H., Zhao, Y., Wang, P.: Solubility determination and thermodynamic modeling of n-acetylglycine in different solvent systems. Journal of Chemical & Engineering Data **66**(3), 1344–1355 (2021)

[10] Ross, J., Belgodere, B., Chenthamarakshan, V., Padhi, I., Mroueh, Y., Das, P.: Large-scale chemical language representations capture molecular structure and properties. Nature Machine Intelligence **4**(12), 1256–1264 (2022)

[11] Ahmad, W., Simon, E., Chithrananda, S., Grand, G., Ramsundar, B.: ChemBERTa-2: Towards Chemical Foundation Models (2022). https://arxiv.org/abs/2209.01712

[12] Boobier, S., Hose, D.R., Blacker, A.J., Nguyen, B.N.: Machine learning with physicochemical relationships: solubility prediction in organic solvents and water. Nature communications **11**(1), 5753 (2020)

[13] Vermeire, F.H., Chung, Y., Green, W.H.: Predicting solubility limits of organic solutes for a wide range of solvents and temperatures. Journal of the American Chemical Society **144**(24), 10785–10797 (2022) https://doi.org/10.1021/jacs.

2c01768

[14] Cihan Sorkun, M., Mullaj, D., Koelman, J.V.A., Er, S.: Chemplot, a python library for chemical space visualization. Chemistry-Methods **2**(7), 202200005 (2022)

[15] Krasnov, L., Mikhaylov, S., Fedorov, M., Sosnin, S.: Bigsoldb: Solubility dataset of compounds in organic solvents and water in a wide range of temperatures. ChemRxiv (2023) https://doi.org/10.26434/chemrxiv-2023-qqslt
